# Key factors in the future of oral and dental health in Iran using scenario writing approach

**DOI:** 10.1186/s12903-024-04354-y

**Published:** 2024-05-14

**Authors:** Mohammad Hossein Mehrolhassani, Mostafa Mozhdehifard, Rohaneh Rahimisadegh

**Affiliations:** 1 Health Services Management Research Center, Institute for Futures Studies in Health, Kerman University of Medical Sciences, Kerman, Iran; 2 Department of Health Management, Policy and Economics, Faculty of Management and Medical Information Sciences, Kerman University of Medical Sciences, Kerman, Iran

**Keywords:** Future studies, Scenario writing, Oral health, Dental health, Key factors, Iran

## Abstract

**Background:**

Oral and dental health can significantly impact individuals’ quality of life. The World Health Organization introduces oral health as one of the essential priorities of public health worldwide. Given the lack of studies on the future of oral and dental health in Iran, this study used a futures studies approach to identify the factors in oral and dental health in Iran through scenario writing.

**Methods:**

This study was conducted in three stages including the scenario writing approach, qualitative methods, and exploratory future research. First, potential variables affecting future oral and dental health systems were extracted through interviews. The focus group discussion determined the uncertainty and importance of the variables. Then, the cross-impact balance matrix was imported into the Scenario Wizard software to identify the different states of the scenario generator variables and compatible scenarios were extracted.

**Results:**

Seventy variables were extracted as key variables affecting the future of oral and dental health. Regarding the importance and uncertainty, seventeen variables scored higher and fell into policy and governance, economy and financing, social, service delivery, and technology, serving as five categories of scenario generators. Fifteen scenarios with weak consistency and three with strong consistency were obtained using the Cross-Impact Balance matrix in Scenario Wizard software.

**Conclusion:**

The probability of a pessimistic scenario where all five categories of the scenarios were in the worst possible state was higher due to its consistency. The government’s support policies and commitment to oral and dental health were two key factors in the future. Achieving an optimistic and favorable scenario for the future of the country’s oral and dental health system depends on the government and policymakers in the health sector adopting a positive attitude towards the role of oral and dental health services in improving societal health. In this scenario, the five categories of the scenario generators were in the best condition.

**Supplementary Information:**

The online version contains supplementary material available at 10.1186/s12903-024-04354-y.

## Background

The impact of oral and dental diseases on individuals’ daily quality of life is undeniable. For this reason, the World Health Organization(WHO) introduces oral and dental health as one of the most critical public health priorities worldwide [1, 2]. However, this issue is still a public health problem even in some developed countries and developing countries [3]. So far, many countries have failed in preventing oral and dental diseases and improving oral and dental health indicators, and the burden of diseases related to this area is increasing as a silent epidemic [4–6].

Studies on the burden of diseases in 2017 have indicated that oral diseases affect more than half of the world’s population [4, 5].Various studies have confirmed the relationship between oral and dental health and the physical, mental, and social aspects of health, highlighting its importance as one of the essential components of public health [7]. Taking care of oral and dental health is a gateway for early diagnosis and prevention of many oral as well as many systemic diseases [8]. Based on the common risk factors approach, oral diseases are considered a risk factor in developing or increasing the severity of systemic conditions [9]. For example, evidence shows that oral diseases can significantly affect the development of systemic conditions such as cardiovascular diseases, diabetes, various cancers, and cognitive decline [8]. Additionally, oral diseases can lead to other complications, such as swallowing, chewing, as well as speech problems, impacting sleep quality and productivity [10].

Many factors can contribute to oral and dental diseases, including insufficient self-care, lack of access to oral and dental healthcare services, socioeconomic factors, and personal problems [11, 12]. The policies adopted in oral and dental health can significantly improve the oral and dental health by ensuring access to health care services and addressing the various challenges associated with oral and dental health [13]. Different policies implemented in oral and dental health lead to various executive approaches due to the country’s different political and social conditions [14]. While tooth decay is overlooked by health policymakers in many countries as one of the most common oral and dental health issues [15, 16], oral and dental diseases are recognized as significant factors by policymakers, individuals, and communities in other countries [17]. Given the aging population as one of the leading global trends, the policies adopted regarding routine dental examinations and dental treatments for the elderly plays a crucial role in preventing health issues, improving the quality of life, and reducing healthcare costs worldwide [18]. The number of elderly individuals in society is steadily increasing. With the rise in the elderly population, oral and dental problems such as tooth loss, root caries or periodontal problems, denture issues, oral cancer and so on, have become more prevalent [19]. All these conditions can compromise older people’s quality of life [20]. Thus, tailored policies for older adults need to be developed considering the need and changes in oral health diseases of this population [21].

Formulating and implementing effective dental and oral health policies can be challenging in Iran because of low dental budget allocation and inappropriate development model [13]. Achieving the optimal status of oral and dental health indicators is a great economic burden. This issue, along with ignoring oral and dental health at the policy level and upstream documents of the country, has had a double impact on oral and dental health [4, 22]. In Iran, medical services, including dental services, are mainly provided by three basic insurance funds, including Iran Health Insurance Organization (IHIO), Social Security Organization (SSO), and Armed Forces Medical Services Insurance (AFMSI). All three basic insurance funds are required to cover oral and dental health services such as dental checkups, periapical/bitewing radiography, tooth extractions, surgical removal of impacted and semi-impacted teeth, subgingival scaling (only for individuals older than 12 years), dental prophylaxis, and fissure sealant for the first and second permanent molars as part of the essential oral and dental health care package services [23]. This is while basic insurances pay 3% of the costs of services such as scaling, simple radiography, surface filling, and tooth extraction, and supplementary insurance companies bear 7% of dental costs, and other costs are paid out of pocket by patients [13]. The Ministry of Health and Medical Education (MOHME), as the main provider of public services, provides some oral and dental health services covered by basic health insurance, mainly in rural areas [24].On the other hand, the majority of dental services are provided by the private sector, while almost 90% of dentists work in urban areas [25, 26]. Recently, the MOHME has made efforts to increase the coverage of dental services; however, it has not entered the implementation phase [27]. Providing fair access to dental services in Iran has not received as much attention as it should, and policies must be reviewed to provide fair, efficient, and effective dental services in the future [28].

Given the status quo, policymakers and managers in the health field should take a practical step in implementing appropriate strategies in preventive and therapeutic policies for oral and dental health systems in the future. Considering the lack of studies on the future of Iran’s oral and dental health system, it is necessary to conduct future studies in this regard. Future research helps managers and policymakers make decisions for the future in uncertain conditions [29]. Therefore, this study was conducted using the future research approach and the scenario writing method in response to the question, “What are the key and scenario-creating factors in the formation of the future of oral and dental health in Iran, and what are the possible futures based on them?” The results can help managers and policymakers of the health system, especially in oral and dental health, in planning and making appropriate and timely decisions to face challenges and improve the future oral and dental health situation.

## Methods

### Study design

**Fig. 1:**
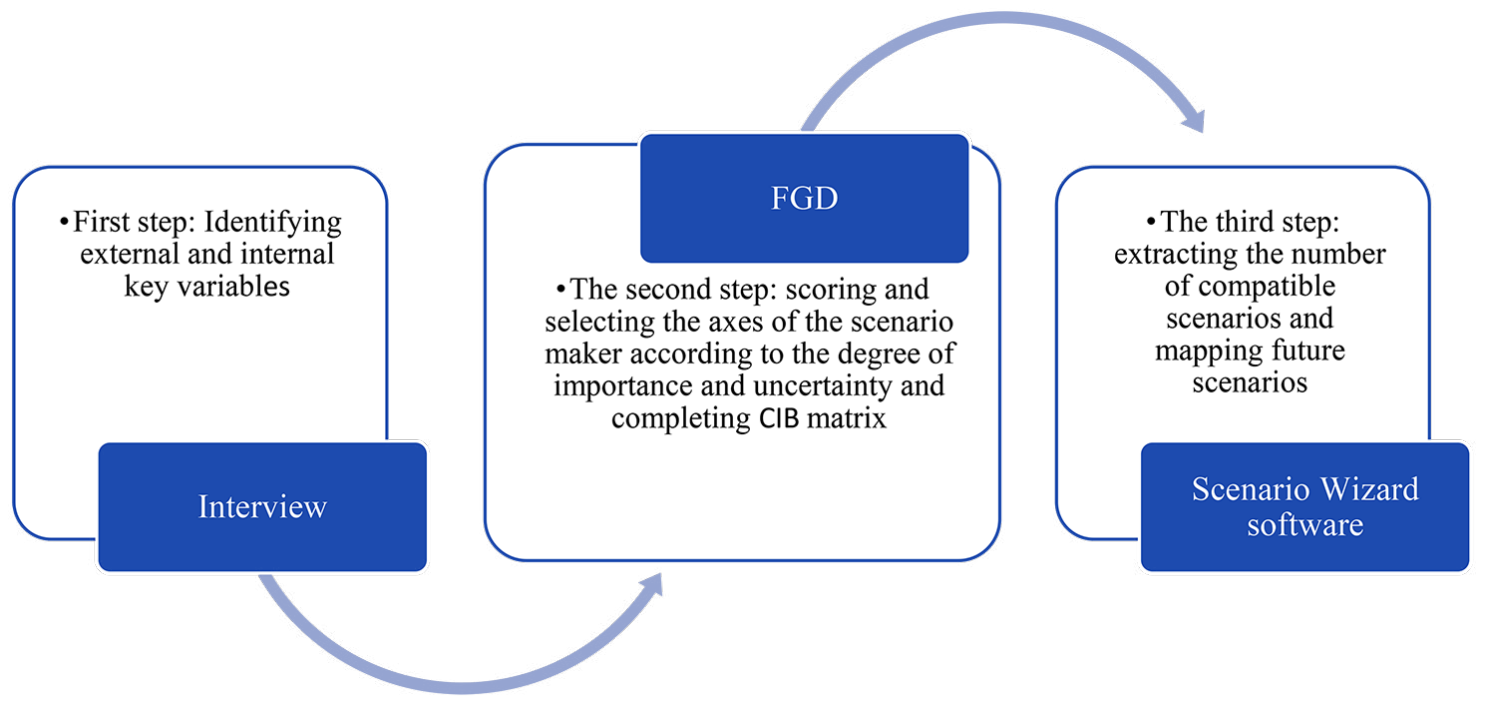
Steps of study

Three steps of scenario writing approach, qualitative methods, and exploratory future studies were used in this study (Fig. 1). In the first step, experts were interviewed to identify the key internal and external factors influencing oral and dental health in the future. As part of the second phase, a focus group discussion (FGD) was conducted to identify the essential variables from the perspectives of importance (influence) and uncertainty (probability of occurrence). Then, the Cross-Impact Balance (CIB) analysis was completed to identify the different states of the scenario variables. In the third step, the number of compatible scenarios was extracted by entering the CIB matrix into Scenario Wizard Software, and finally, the future scenarios were compiled.

### Study method

Scenarios paint images of possible futures. The critical point is that a scenario is not a comprehensive image of the future, but rather a way to direct attention to one or more specific and clear parts of reality in future. Therefore, in our case, the set of key variables for the correct description of each system and the interactions between them that shape the future should be investigated in order to explore the system’s behavior in the future. A wide variety of methods are available to design scenarios. CIB analysis is one of the most well-known methods, and it seeks to find direct and indirect relationships between variables by relying on experts’ decisions to structure and formalize judgmental prediction [30].

Scenario Wizard is an application software in scenario writing that uses the CIB method to evaluate potential future scenarios and facilitate qualitative information processing and expert opinion analysis. The scenario wizard is the most widely used application in future research based on the CIB matrix as verbal expressions to extract experts’ opinions about the probability of a state of one descriptor on another state of the descriptor by calculating the direct and indirect effects, which are stated on top of each other. Compatible scenarios are extracted in advance on the studied system [31]. The scores that the experts give to the different states of the descriptors in the CIB matrix are based on their opinions [32]. Table 1 lists the meaning of scores.

**Table 1:** Scoring in the CIB matrix

Qualitative scale of judgment of experts	Meaning of score
-3	strongly restricting influence
-2	moderately restricting influence
-1	weakly restricting influence
0	no influence
+ 1	weakly promoting influence
+ 2	moderately promoting influence
+ 3	strongly promoting influence

### Participants

The participants were recruited based on purposive sampling. In the first stage, the inclusion criterion was at least five years of scientific and executive experience in oral and dental health at the national and provincial levels. The interviewees were purposefully selected among managers, policymakers, experts in oral and dental health, and faculty members of medical sciences universities. In the second stage, the inclusion criterion was at least 10 years of scientific and executive experience in oral and dental health. These participants were purposefully selected among managers, policy makers, experts in oral and dental health, and faculty members of medical sciences universities. The characteristics of all participants in the two stages of the study are listed Table 2.

**Table 2:** Characteristics of participants in interview and FGD

General category	Characteristic	No. in interview	No. in FGD
Organizational position	Expert	15	1
	Manager/policy maker	14	5
	Faculty member	2	2
Geographical scale	Provincial	14	5
	National	17	3
Level of Education	Specialization - subspecialty	9	5
	General Dentist - PhD	7	3
	General Dentist	14	-
	Masters	1	-
years of experience	Less than 10 years	3	-
	10 to 20 years	12	5
	20 to 30 years	14	3
	More than 30 years	2	-
Total		31	8

### Data collection

Semi-structured interviews were used to collect data (Appendix 1). The interviews were arranged in advance and conducted in person at the participants’ workplaces. The consent for recording was obtained from the participants before performing the interview. The main interview question was about the key factors affecting the oral and dental health system. The researcher recorded all the interviews and notes were taken of essential points during the interview for further clarification. On average, the interviews took 20 min. A focused group discussion was formed in the second step. The experts were asked to express their opinions quantitatively by marking an Excel form in order to specify the degree of uncertainty and importance of each identified variable in the previous step. Next, the CIB matrix was used to score the different states of the categories of the scenario creator. Finally, the Scenario Wizard software (version 4.31, Wimer, the University of Stuttgart, Germany) was used to find the number of compatible scenarios. The scenario writing method illustrated the future of oral and dental health in Iran.

### Data analysis

The thematic analysis method was used to analyze the interviews and identify factors affecting the future of oral and dental health. The key factors influencing the oral and dental health systems were divided into two categories, external and internal factors, according to the boundaries of the health system. External factors refer to factors outside the organization’s influence and control, and internal factors represent the subsystems that make up the organization’s system and have a direct and consequential effect on the organization’s activity and performance level [11]. The key factors within the health system were categorized into six categories (governance and leadership, financing, human resources, medicine and equipment, health information systems, and service delivery) based on the WHO structural blocks model [33].The key factors outside the health system were classified into five categories (Social, Technological, Environmental, Economic, and Political) based on the STEEP model [34]. In the second step, the categorized factors were entered into an Excel Spreadsheet database, and the importance and uncertainty score of each variable were scored from 1 to 10 by the FGD participants. According to the experts, the variables with an importance score above seven and uncertainty above four were selected as scenario generator variables. The effectiveness and influence of each scenario variable on each other in the CIB matrix were determined numerically between positive three and negative three. Positive three indicates a positive effect and a high strengthening property of one variable compared to another. Negative three indicates an adverse effect and inhibitory property of one variable compared to another. A zero number shows that the two variables are neutral to each other.

The CIB matrix was imported into the Scenario Wizard software to analyze the matrix with all possible modes to predict the variable placement based on mathematical models. The essential feature of this software is extracting the most compatible scenario mode based on countless modes [35]. The number of solid scenarios that were more likely to occur was achieved because of the high consistency of the conditions. Future scenarios were developed by using different states of the categories of the scenario generator.

## Results

Seventy variables (including 43 internal and 27 external variables) were identified as critical factors influencing oral and dental health in the future. Seventeen variables were selected as scenario generator variables in five scenario generator categories namely politics and governance, economy and financing, social, service delivery, and technology (Table 3) based on the FGD participants’ opinions.

**Table 3:** The average importance and uncertainty of scenario variables based on the results of FGD

Axis of the scenario generator	Scenario generator variables	Average importance	Average uncertainty	External/internal
Politics and governance	Government support policies	9.3	4.9	external
	Government obligations	9.3	5.0	external
	The structure of the country’s political system	8.2	4.8	external
	Understanding the importance of oral health by health policy makers	9.2	5.7	internal
	Service delivery structures	8.9	4.8	internal
	Efficient and transparent laws, policies and processes	8.2	5.0	internal
	The responsible of the government in planning and providing services	8.0	5.6	internal
	Decision-making structures in different fields	8.5	4.4	internal
	Designing and organizing preventive programs	8.2	5.5	internal
	Interaction between the whole health system and oral health system	8.3	5.7	internal
Economics and financing	Government budget and credits	8.7	5.9	external
	Family economic	8.2	5.3	external
	Allocation of financial resources of oral health system	8.2	5.2	internal
	Insurance coverage for oral health services	7.8	5.4	internal
Social	The level of health literacy of the community	8.2	4.9	external
Service delivery	Commercialization and merchandising of providers	8.1	4.9	internal
Technology	Knowledge-based production of dental raw materials in the country	7.0	5.0	external

**Fig. 2:**
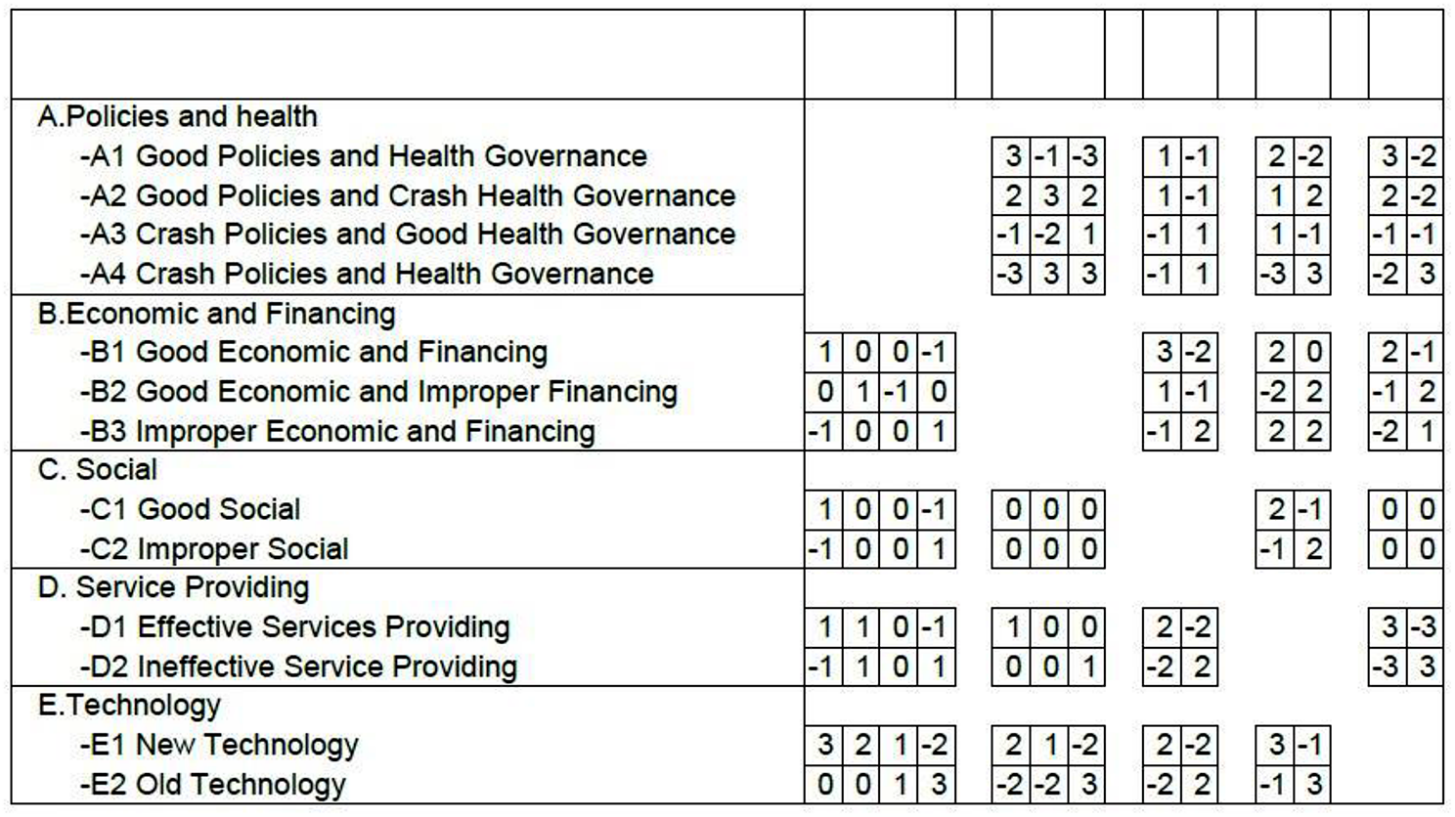
The CIB matrix entered into the scenario software

**Fig. 3:**
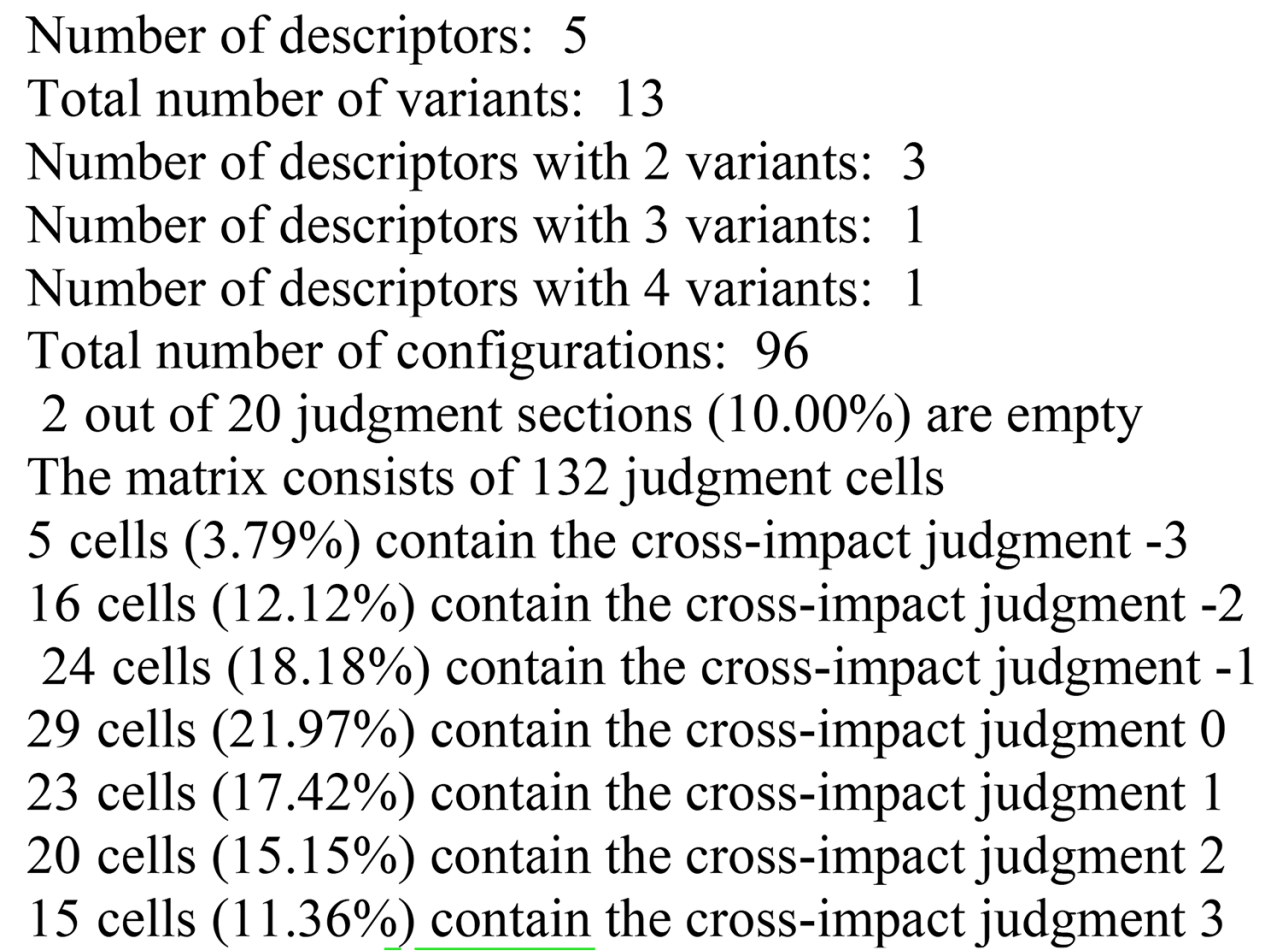
Overall project information

Every research team member completed the 13$\times$13 CIB matrix, which was developed in Excel for the five scenario generator categories based on their various states. A consensus amongst divergent researcher opinions was reached in a joint meeting, and the CIB matrix was used as an input for entering into the Scenario Wizard program (Fig. 2). Figure 3 displays the overall project details shown in the scenario wizard program.

**Fig. 4:**
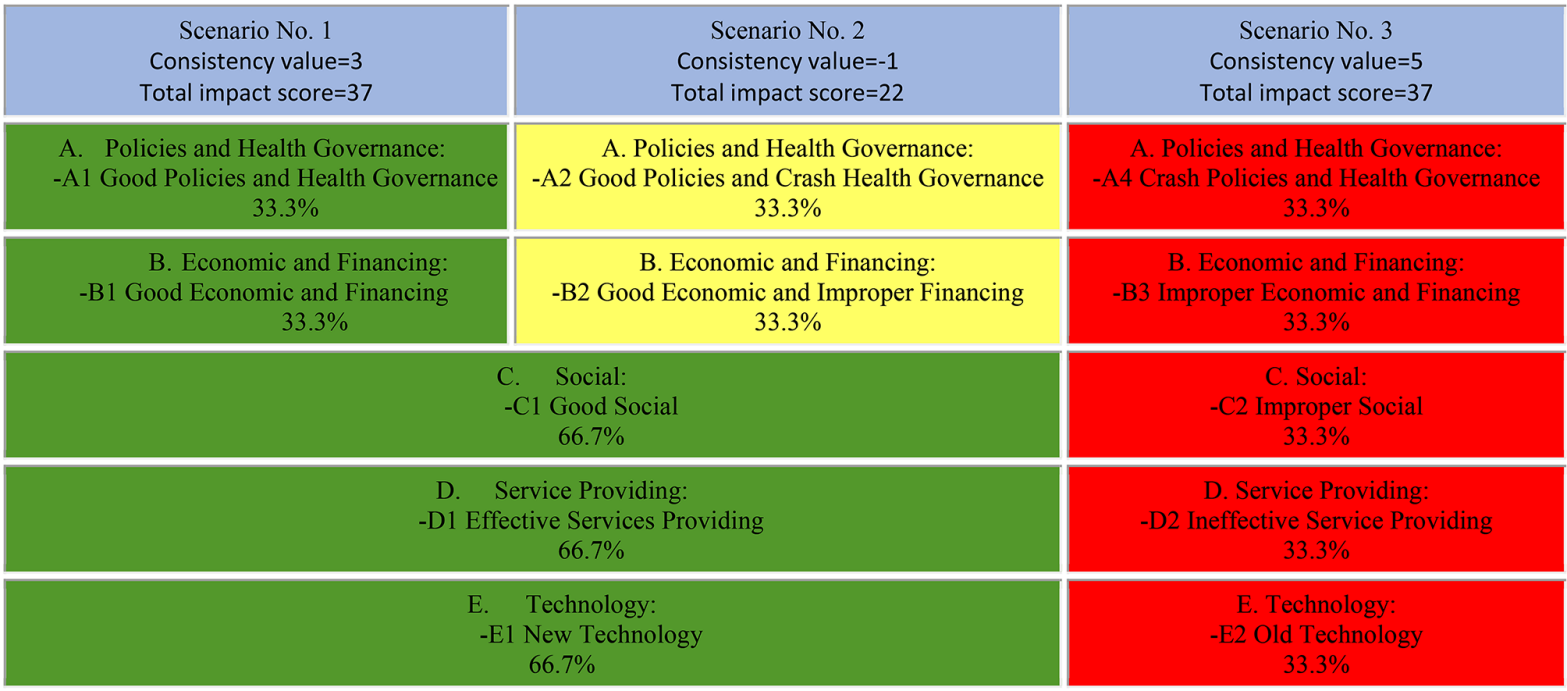
Three scenarios with high consistency and strong probability of occurrence for the future of oral and dental health in Iran

In the third step, 15 weak and two robust probability scenarios were identified based on the information output of the Scenario Wizard software. In order to increase the number of robust scenarios, max. inconsistency figure in scenario wizard software was set at 1 the first time and 2 the second time. As a result, the number of solid scenarios with high consistency increased to 3, as shown in Fig. 4. This figure displays the best possible states for each category in green, while the intermediate and worst possible states are in yellow and red, respectively. There was a 53% probability of the best state (green color), a 13% probability of an intermediate category state (yellow color), and a 34% probability of the worst category state (red color) in the future. Aside from the percent probabilities generated, Scenario Wizard displays the best category state (green color), intermediate category state (yellow color), and worst category state (red color). For example, the probability of good social conditions in three robust scenarios is 66.7%, while the second stage of this category has a probability of 33.3% under unfavorable social conditions.

## Discussion

Multiple factors affect the future of oral and dental health in Iran. Considering these factors, five categories—policy and governance, economy and financing, social, service delivery, and technology—were identified as scenario generator categories with different states. As a result, three scenarios with a higher probability of occurrence were developed for the future of oral and dental health, and each of the axes was discussed in the scenario space.

### Politics and governance category

In the first scenario, the structure of the country’s political system is such that the government considers itself committed to providing oral and dental health services and shows its support by adopting clear policies and laws. The policymakers of the health system understand the importance of oral and dental health in promoting the health of society. Therefore, the health system trustees implement laws and policies supporting oral and dental health in line with the government’s policies. The health system trustees establish a good interaction and cooperation between the entire health system and the oral and dental health system to design preventive oral and dental health programs. This will be in spite of multiple decision-making structures at the macro level of the country and the level of the health system. In the second scenario, the government is committed to providing oral and dental health services; however, policymakers and health system management do not understand its importance well. In the third scenario, oral and dental health does not have an important position among the health system’s managers and policymakers, and there is not much-related support available.

Despite the preventable nature of a significant portion of oral diseases, health policymakers, and officials often do not understand the importance of primary oral and dental health. International policies support integrating oral and dental health policies into all health policies and emphasize the interaction of oral and dental health professionals with health leaders and policymakers at the governmental and non-governmental levels -i.e. local, regional, national, and global level [2]. Molete et al. revealed that the managers’ lack of understanding of the policies at the local level is one of the main challenges in implementing oral and dental health policies, which originates from a lack of common language between policymakers and managers [36]. Tahani et al. showed that Iran’s health system needs to improve the overall performance of oral and dental health system management and policymaking as one of its sub-systems [37], which is essential in advancing and enhancing the oral and dental health system. The decisions made at the level of councils and macro-policy committees of the country play a crucial role in ensuring better and more sustainable oral and dental health for all members of society. Anyone at any income level at any geographic location (city and village) can access oral and dental services through these decisions [38].

### Economic and financing category

The first scenario of Iran’s oral and dental health system is one in which the government allocates reasonable budgets for the country’s health sector, resulting in financial resource stability which is needed for oral and dental health, particularly in prevention and health services. In the health sector, stable and significant financial resources are provided to the oral and dental health, especially in prevention and health services. Secured budget can ensure free oral health care through preventive health services. Dental treatments will be covered by insurance, so that essential services are covered by basic insurance and non-essential dental treatments are covered by supplementary and private insurances. Families can purchase services not covered by insurance. The government allocates funds and credits to preventive oral and dental health services in the second scenario; however, financial resources within the health system are unevenly distributed. Insurance does not cover many services, such as dental treatments, which imposes a significant financial burden on society. Since the households are not in good economic condition, they are exposed to catastrophic health expenditures. In the third scenario, a situation like the second scenario can occur when the government fails to allocate proper budgets and credits for preventive oral health services.

Oral and dental diseases are among the most common diseases worldwide, which impose a significant economic burden on governments. Currently, the cost of dental treatments in the EU countries is in third place, with 90 billion euros compared to 111 billion euros for diabetes and 119 billion euros for cardiovascular diseases. Despite the allocation of sufficient financial resources to oral and dental health by governments, this has not necessarily led to better outcomes in the realm of oral health and dental care or increased financial protection for the populace. The point is whether these allocations are directed towards the procurement of therapeutic services or preventive care services [39].

Based on the results of studies, families in Europe not in good economic conditions suffer from oral and dental health problems more than other families. Economic inequality prevents access to dental services and it is considered a significant challenge for the healthcare system. Likewise, reducing the burden of oral and dental health problems in low-income families imposes a significant financial burden on the healthcare system [40]. Fair access to oral and dental health services potentially reduces total health costs [26].

In economic crises, people’s purchasing power decreases drastically due to increased financial pressure and inflation. Therefore, families plan to reduce the quantity and quality of goods and services and implement strategies to reduce costs in all sectors, including the health sector. In this situation, some health services, such as dental treatments, become a privilege inaccessible to working and middle class families in such societies [41].The decline in access to dental treatments is mainly because these services are often considered unnecessary and very expensive [39], or if they do purchase dental treatments, they are exposed to catastrophic health costs. Studies have indicated that people in many countries should pay a significant portion of their dental treatments expenses out of pocket, leading to catastrophic health expenditures in families [42]. In addition, studies have shown that people in Iranian households who use dental treatments have a higher likelihood of experiencing catastrophic health expenditure [26]. In Iran, the private sector provides more than 80% of the country’s dental treatments, and more than 90% of dental treatments costs are paid out of pocket because insurance companies do not cover much due to the limited services covered [43]. Therefore, the government’s insurance coverage of dental treatments is necessary to prevent catastrophic health expenditures for families with a low economic status [44].Financing the health system in Iran with insurance comes with challenges that also affect the oral and dental health systems. The complex system of collecting income and the multiplicity of insurance funds [45] presents another obstacle to using insurance, affecting the oral and dental health system. The lack of strategic purchase of oral and dental health services is another significant challenge, as these services are purchased passively and non-strategically [22].The solution to the first fundamental challenge is the consolidation of insurance funds [46]. The primary benefits package of oral and dental health services needs to be designed to provide services with a greater focus on prevention in order to improve the strategic purchasing process based on factors such as the relative cost-effectiveness of interventions and the burden of diseases [22]. In recent years, in many countries, economic crises and financial pressures on governments have affected the insurance coverage of dental treatments and the definition of benefits packages, so that the insured services have decreased and the benefits packages have become more limited day by day [47]. Patients’ treatment choices are strongly affected by high dental treatment costs, and their economic status [48]. This has led to around 3% of the European population not receiving dental treatments for financial reasons [47] and individuals with lower income do not visit dentists until they have to receive emergency treatment [48].

### Social category

The community has a high level of health literacy regarding oral and dental health in the first and second scenarios. As a result, society maintains a positive attitude towards preventive oral and dental health care and embraces suitable lifestyle and nutritional patterns at different stages of life. In the third scenario, the community’s literacy level regarding oral and dental health is low. For this reason, the community does not have a positive attitude towards preventive oral and dental health care, and they use more dental treatments.

Health literacy is one factor affecting oral and dental health, effectively creating more or fewer costs for the health system. Literacy in health refers to the ability to access, comprehend, and use health-related information and services to promote and maintain health [49]. The high health literacy level of individuals regarding dental hygiene leads to the improvement and promotion of the oral and dental health of society. Some argue individuals with low health literacy often have poor health status, unhealthy behaviors, and less use of preventive services, and they have higher rates of chronic diseases and higher health care costs than individuals with higher health literacy levels [50]. In addition to the literacy level of society, people’s lifestyles also have a significant impact on the future of the dental health system. Today, major oral and dental diseases, like other non-communicable diseases, are considered lifestyle-related conditions, and it is expected that with the increase in health literacy levels and changes in lifestyle and health behaviors, individuals will play a more active and effective role in maintaining, improving, and promoting their own health. Recent scientific findings support the fact that with the increase in society’s understanding and literacy about oral health, the health status of individuals in other diseases also improves [5], as oral diseases share similar risk factors with other diseases [51]. In addition, dental teams can play a fundamental role in providing a positive model of health-promoting behaviors through their professional knowledge in the field of preventive interventions [9].

### Health service delivery category

In the first and second scenarios of the future oral and dental health system, providers deliver oral and dental health services according to the principles of professionalism and prioritize health and preventive services. Service delivery mechanisms are formed using the design and organization of preventive programs to provide the best oral and dental health services. In the third scenario, profit-seeking and speculation replace professional principles. In this scenario, the field of oral and dental health becomes a money-making industry and business for providers of expensive and luxury unnecessary and super-specialized medical services with a demand approach.

Commercialization and profiteering of oral and dental health service are a direct threat to professional values and patient care [52]. There are many contradictions regarding professionalism and commercialism in providing dental care among the providers, i.e. dentists. Some dentists consider the provision of dental care equal to the provision of medical services and the work of doctors and describe oral and dental health services as essential. On the other hand, some dentists consider dental services a commercial service and define the services of this field as luxury services [53]. Their advertisements only promote luxury and unnecessary dental services, ignoring the importance of preventive care and health [54]. This attitude makes it difficult for people who do not have a good financial situation to access essential oral and dental health services. According to the principles of professionalism, oral and dental health providers should resist turning services into a commercial and profit-seeking factor and instead focus more on offering health and preventive services. This issue (the lack of primary oral and dental health care and an inappropriate approach to preventive dental care), is a common concern of policymakers and governments in many countries [10]. Despite the increase in demand for luxury dental care from consumers, dentists should know how the attitude of profiteering and greed among them may harm oral and dental health and the public trust in this profession [53].

### Technology category

In the first and second future scenarios for Iran’s oral and dental health system, new technologies will revolutionize the field, and localized production of dental materials will occur. In the third scenario, contemporary oral and dental health technologies are imported from advanced countries, and we still rely on other countries for dental materials.

Science and technology are rapidly advancing, leading to new technologies in oral and dental health. Dental technology includes various fields involved in the manufacturing of dental prostheses, such as veneers, implants, bridges, and artificial teeth for functional and aesthetic purposes. Dental technology has undergone significant changes in recent years because of technological advancements. Among others, one development that attracted considerable attention is integrating artificial intelligence in dentistry [55]. The application of artificial intelligence technology and machine learning in various fields of oral and dental health, such as radiographic interpretation, diagnosis, and dental and maxillofacial surgery, has revolutionized the dental profession [56]. Expanding the use of digital health technologies, especially mobile digital health technologies, and other emerging technologies in the future, will promote better and greater access to the knowledge of oral and dental health professionals for urban and remote populations in developed and developing countries [2]. In addition, digital health technologies can improve the health literacy of community members regarding oral hygiene and can advertise and promote a variety of appropriate interventions necessary in different periods of life, such as special education programs for children, mothers, and older adults [57]. In addition, new knowledge and technologies can potentially create tremendous changes in diagnosing and managing oral and dental diseases [8, 58]. In addition, nanotechnology in dentistry has received serious attention in recent years [59], and nanotechnology is practical in various fields of dentistry, from diagnosis to prevention and treatment [60]. Nanotechnology has created a new perspective on traditional materials and methods. The benefits and effectiveness of this technology in dentistry shows that the widespread use of this technology in oral and dental health is promising [59]. New technologies to treat oral diseases increase patients’ satisfaction and the quality and durability of the performed treatments, reduce the length of treatment processes, and decrease unwanted side effects [59].

This study was conducted before the COVID-19 pandemic, so the experts’ ideas about what would affect oral and dental health in the future might be slightly different now.

## Conclusion

Many internal and external factors influence the oral and dental health system, some of which are controllable and some are beyond the control of the managers and policymakers of the health system. The future of dental and oral health differs significantly from today due to many changes in today’s economic, social, and political fields. Therefore, it is necessary to start thinking about the future today by using future research techniques such as scenario writing and drawing futures. The results indicated that several factors shape the future of oral and dental health in Iran, including politics and governance, economy and financing, social, service provision, and technology. Accordingly, researchers identified three scenarios with a strong likelihood of shaping the future of the oral and dental health system. Two key factors were the government’s commitments and support policies towards oral and dental health, as identified in the study. Similarly, the government and policymakers in the health sector should have a positive attitude and vision regarding the importance and role of primary oral and integration of primary dental health services in the whole health system, which is an investment to improve people’s overall health. The future of oral and dental health may move toward a favorable scenario (the best scenario of the scenario maker’s categories). In contrast, without such support the possibility of an unfavorable and pessimistic scenario (the worst case of the scenario maker’s categories) is more likely to occur. Given the current situation, this study demonstrates the expected unfavorable national oral and dental health in future. Now, it is time to make a decision for a better oral and dental health future by all parties involved.

### Electronic supplementary material

Below is the link to the electronic supplementary material.

Supplementary Material 1

## Data Availability

The data analyzed is available from the corresponding author on reasonable request.

## Abbreviations

WHO: World Health Organization
FGD: Focus Group Discussion
CIB: Cross-Impact Balance
STEEP: Social, Technological, Environmental, Economic and Political
MOHME: Ministry of Health and Medical Education
