# Supplementary material for: Key factors in the future of oral and dental health in Iran using scenario writing approach
Source: BMC Oral Health. 2024 May 14;24:563. doi: 10.1186/s12903-024-04354-y (PMC11092190; doi:10.1186/s12903-024-04354-y)
Supplement: Supplementary file 1 — Supplementary Material 1 [file 12903_2024_4354_MOESM1_ESM.docx]

***Appendix 1 :Interview framework***

**Interviewer information:**

First and last name:

Organizational position:

Geographical scale:

Level of Education:

years of experience:

**In your opinion:**

1. What are the internal key factors in the Future of Oral and Dental Health in Iran?
2. What are the external key factors in the Future of Oral and Dental Health in Iran?
